# A Genome-Wide Association Study Identifies Genetic Variants Associated with Mathematics Ability

**DOI:** 10.1038/srep40365

**Published:** 2017-02-03

**Authors:** Huan Chen, Xiao-hong Gu, Yuxi Zhou, Zeng Ge, Bin Wang, Wai Ting Siok, Guoqing Wang, Michael Huen, Yuyang Jiang, Li-Hai Tan, Yimin Sun

**Affiliations:** 1 Center for Neurogenetics, Shenzhen Institute of Neuroscience, Shenzhen, 518057, China; 2 State Key Laboratory of Proteomics, Beijing Proteome Research Center, Beijing Institute of Radiation Medicine, Beijing, 102206, China; 3 Department of Healthy Management, Research Institute of Surgery, DaPing Hospital, Third Military Medical University, Chongqing, 400042, China; 4 CapitalBio eHealth Science & Technology (Beijing) Co., Ltd., Beijing, 102206, China; 5 National Engineering Research Center for Beijing Biochip Technology, Beijing, 102206, China; 6 Department of Linguistics, The University of Hong Kong, Hong Kong, China; 7 Department of Anatomy, The University of Hong Kong, Hong Kong, China; 8 The State Key Laboratory Breeding Base-Shenzhen Key Laboratory of Chemical Biology, The Graduate School at Shenzhen, Tsinghua University, Shenzhen, 518055, China; 9 School of Biomedical Engineering, Shenzhen University Health Science Center, Shenzhen, 518060, China; 10 Department of Biomedical Engineering, Medical Systems Biology Research Center, Tsinghua University School of Medicine, Beijing, 100084, China

## Abstract

Mathematics ability is a complex cognitive trait with polygenic heritability. Genome-wide association study (GWAS) has been an effective approach to investigate genetic components underlying mathematic ability. Although previous studies reported several candidate genetic variants, none of them exceeded genome-wide significant threshold in general populations. Herein, we performed GWAS in Chinese elementary school students to identify potential genetic variants associated with mathematics ability. The discovery stage included 494 and 504 individuals from two independent cohorts respectively. The replication stage included another cohort of 599 individuals. In total, 28 of 81 candidate SNPs that met validation criteria were further replicated. Combined meta-analysis of three cohorts identified four SNPs (rs1012694, rs11743006, rs17778739 and rs17777541) of *SPOCK1* gene showing association with mathematics ability (minimum p value 5.67 × 10^−10^, maximum β −2.43). The *SPOCK1* gene is located on chromosome 5q31.2 and encodes a highly conserved glycoprotein testican-1 which was associated with tumor progression and prognosis as well as neurogenesis. This is the first study to report genome-wide significant association of individual SNPs with mathematics ability in general populations. Our preliminary results further supported the role of *SPOCK1* during neurodevelopment. The genetic complexities underlying mathematics ability might contribute to explain the basis of human cognition and intelligence at genetic level.

Mathematics serves as a fundamental instrument in modern society as it plays an important role in many fields including science, engineering, and economics. It also used as a key index of human intelligence. Exceptional mathematics ability was frequently observed among genius from many domains. Meanwhile, dyscalculia, characterized by impaired number processing skills, is a specific developmental disorder of mathematics ability that affects approximately 3 to 6% of children^1^. Childhood mathematics ability was associated with adult socioeconomic status and quality of life^2^. Understanding mathematics ability is an essential step to improve children’s numeracy skills and academic achievements and could also provide novel insights into human brain functions. Mathematics ability is a complex trait that involves neurological and cognitive development as well as postnatal education and training. In particular, it is estimated that considerable proportion of variation in mathematic ability could be explained by genetic factors.

Recent years, genome-wide association study (GWAS) has been widely applied to investigate genetic components underlying complex traits^3^. The first GWAS of mathematics ability was performed among children with high and low mathematics ability respectively and nominated top-performing SNPs for subsequent validation in a large sample of individuals spanning the entire distribution of mathematical ability^4^. The study did not observe any SNPs alone showing genome-wide significant association with mathematic ability but hypothesized that genetic contribution to mathematics ability might be explained by multiple quantitative trait locus (QTLs) of small effect^4^. Indeed, the top 10 candidate SNPs only accounted for 2.9% of phenotypic variance in mathematics ability^4^. The second GWAS of mathematics ability used children’s verbal ability as control and then divided them into groups of high and low mathematic ability^5^. Candidate SNPs from the discovery stage were individually genotyped for validation but none of them exceeded threshold of genome-wide significance^5^. In the meantime, another GWAS in monozygotic and dizygotic twin pairs also observed a number of SNPs showing signals of associations with mathematic ability, but none of them achieved genome-wide significant level^6^.

To date, none of studies has identified genome-wide significant association with mathematics ability in general populations due to small effects of common variants. However, research with specific populations might increase statistical power to detect significant association as it was reported that prevalence of mathematic disability was higher among children with neurodevelopmental disorders such as reading disability, attention-deficit/hyperactivity disorder (ADHD) and autism^7^,^8^. The GWAS performed in German dyslexic children identified rs133885 as a genome-wide significant SNP associated with mathematics ability^9^. This variant is a coding variant of *MYO18B* and is associated with intraparietal sulcus morphology^9^. However, a recent replication study of rs133885 failed to find its association with mathematics ability in either dyslexic or general populations^10^.

Previous GWAS of mathematic ability is mainly performed in Western populations of which genetic backgrounds are substantially different to Chinese populations. In the present study, we performed GWAS of mathematic ability in the Han Chinese elementary school students through QTL-based approach. In total, we identified four SNPs exceeding genome-wide significant threshold which is the first time to report genome-wide significant association of individual SNPs with mathematics ability in general populations. Our results provide novel evidence to explain genetic complexities underlying mathematic ability and the basis of human intelligence at the genetic level.

## Results

**Figure 1:**
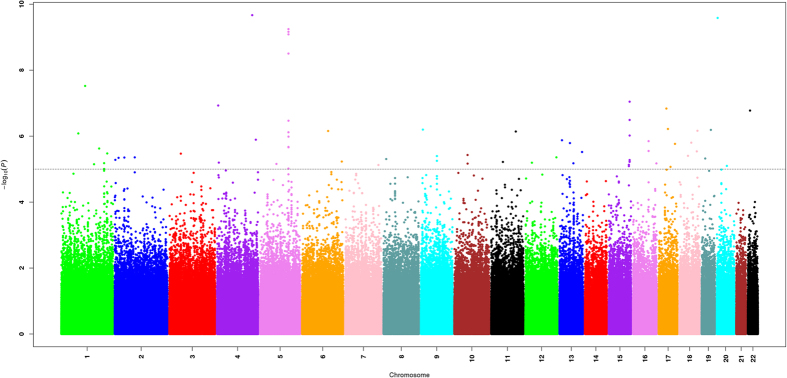
Manhattan plot of –log_10_ (P values) of meta-analysis result from the additive model after adjustment for sex, age, school and nominal significant principal components in GWAS in Liangshan and Dongming population. The genome-wide threshold for significant (P = 5 × 10^−8^) and suggestive (P = 1 × 10^−5^) association are indicated by the horizontal blue and red lines, respectively. 81 SNPs in meta-analysis had P value < 1 × 10^−5^, of which 28 met the criteria for further replication. The symbol for the gene where the significant SNPs are in combined meta-analysis is shown in italics.

In the initial discovery phase, we performed a GWAS scan in two cohorts of Liangshan and Dongming (Supplementary Figure S1; Table 1). Breakdown of math scores according to grades, sex and regions of all participants were presented in Table 2. After quality control, about 1.1 million autosomal SNPs were analyzed (see Methods) in 998 samples (494 from the Liangshan cohort and 504 from the Dongming cohort). We performed linear regression in each cohort with adjustment for age, sex, school and top ten significant principal components of the corresponding cohort to test the additive effect of minor alleles of each SNP. In total, 13082 and 11170 SNPs with P-value less than 0.01 were identified in Liangshan and Dongming cohort respectively (Supplementary Figure S2). Results of the two discovery cohorts were combined by meta-analysis and 81 SNPs with P-value less than 1 × 10^−5^ were identified (Fig. 1). Finally, 28 SNPs met the criteria selection for subsequent replication stage (see Methods; Table 3).

**Figure 2:**
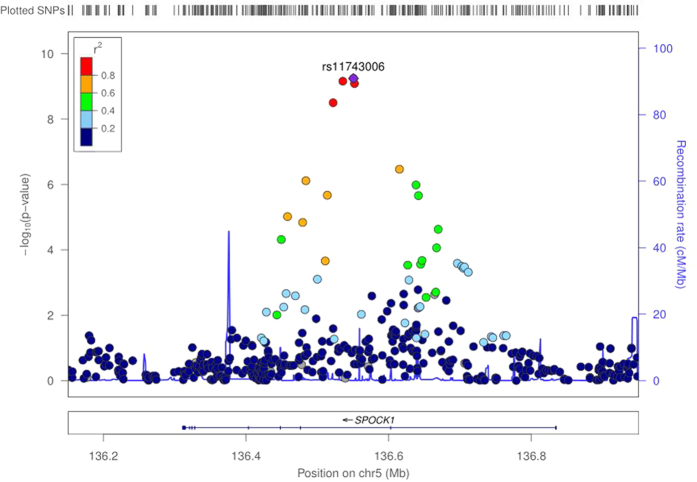
Signal plot of the discovery-stage GWAS meta-analysis for new math score associated locus. Signal plot of GWAS meta-analysis results and recombination rates in the GWAS discovery stage. The results (−log_10_P) are shown for SNPs around the region of *SPOCK1* on chromosome 5. The genes within the region of interest are annotated, and the direction of the transcripts is shown by arrows. The key SNP (rs11743006) are shown in purple and the linkage disequilibrium values (r^2^) for the other SNPs are indicated by the heat scale.

The 28 SNPs met the replication criteria were evaluated in an independent cohort (Cao, Table 4). After meta-analysis of all data from discovery and replication stages, four SNPs (rs1012694, rs11743006, rs17778739 and rs17777541) mapping to *SPOCK1* gene exhibited association on genome-wide significant level for multiple testing (P < 5 × 10^−8^; Fig. 2; Table 4). However, rs17777541 did not show significant association with mathematics ability in the replication population. Results of these four significant SNPs were summarized in Table 5.

## Discussion

Despite substantial heritability underlying mathematics ability, contribution of SNPs to this complex cognitive trait remained inconclusive. Previous GWAS of mathematics ability in general populations proposed candidate SNPs spanning chromosome 2, 3, 4, 5, 6, 7, 11, 12, 13, 20 and 21^4^,^5^,^6^. However, none of them showed consistent association during subsequent replication and therefore failed to exceed genome-wide significant level. Meanwhile, some studies reported sporadic association of copy number variations with mathematics ability but few of them been validated independently^11^,^12^,^13^. In the present study, we performed GWAS of mathematics ability in the Han Chinese general populations. Our genome-wide scan during discovery stage covered all previous nominated regions that might associate with mathematics ability. Although some SNPs located near previous reported region showed significant signal, only four SNPs (rs1012694, rs11743006, rs17778739 and rs17777541) were successfully replicated and achieved genome-wide significant level (minimum P value 5.67 × 10^−10^). Each minor allele of these four SNPs was associated with decrease of math score ranges from 2.33 to 2.43 points approximately. To our knowledge, it is the first time to report association of a single SNP with mathematic ability at genome-wide significant level in general populations.

These SNPs are intron variants of *SPOCK1* and in highly linkage disequilibrium. The most significant SNP rs1012694 is located between exon 3 and 4 of *SPOCK1*. This gene is located on chromosome 5q31.2 and encodes sparc/osteonectin, cwcv and kazal-like domains proteoglycan (testican) 1. Testican-1 is a highly conserved glycoprotein that involved in regulating proliferation, cell-cycle progression, apoptosis, adhesion, and cell-matrix interaction^14^. *SPOCK1* and its gene product testican-1 have been associated with tumor progression and prognosis of different cancer types. Expression of *SPOCK1* at mRNA and protein level was upregulated by the transcription factor CHD1L which could directly bound to promoter region of *SPOCK1*^15^. In hepatocellular carcinoma and gallbladder cancer, elevated expression of *SPOCK1* resulted in activation ofPI3K/AKT signaling which could block apoptosis and promote proliferation, invasiveness and metastasis of cancer cells^16^,^17^. In addition, increased expression of *SPOCK1* was implicated in epithelial-to-mesenchymal transition (EMT) which promoted migration and invasion in lung cancer and esophageal squamous cell carcinoma and conferred acquired drug resistance in gastric cancer^18^,^19^,^20^. Therefore, *SPOCK1* has been considered as a novel prognostic and therapeutic target for various cancer types.

Although the role of *SPOCK1* in cancers has been relatively well understood, its contribution to neurological and cognitive development remains elusive. Recently, novel de-novo *SPOCK1* mutation was reported in a female proband with developmental delay, microcephaly and agenesis of corpus callosum^21^. Her features were similar to previously reported microdeletions of 5q31 for intellectual disability^21^. As there were no mutations or variants of other genes identified in the proband showed potential relevance, *SPOCK1* located within 5q31 was suggested to be a candidate gene of observed developmental abnormalities. The identified de-novo mutation of *SPOCK1* might be protein-damaging which could potentially lead to developmental delay and microcephaly. Therefore, *SPOCK1* might play a critical role during neurogenesis. Indeed, testican-1, encoded by *SPOCK1*, was shown to inhibit attachment of Neuro-2a cells and their ability to form neurite extensions^22^. In addition, it also served as a strong competitive inhibitor of the lysosomal cysteine protease cathepsin L^23^. During early development of mice embryos, testican-1 was strongly expressed in developing brain and modulates neurogenesis and axonal growth^24^. At later developmental stage, testican-1 was particularly prevalent within developing synaptic fields^25^. Altered expression pattern of testican-1 mRNA was observed in reactive astrocytes after brain injury therefore suggested a role of testican-1 in regenerating axons^26^.

Some potential limitations of the present study should be noted. The effect sizes might have been slightly overestimated due to lack of the adjustment for risk factors such as family socioeconomic status. Using nonverbal intelligence as an exclusion criteria might result in biased distribution of children’s math scores as it was assumed that children with lower nonverbal intelligence seems more likely to have lower math scores as well. In addition, sample size of our study were relatively small compared with genome-wide studies of chronic diseases such cancer or diabetes. However, the strengths of our study include its stringent quality control procedures and all participants genotyped by using the Affymetrix Axiom Genome-Wide CHB1 and CHB2 arrays which contain over one million SNPs specifically designed for Chinese population.

In conclusion, we reported four genetic variants of *SPOCK1* that showed genome-wide significant association with mathematic ability in Chinese children. Mathematic ability is a complex trait that involved polygenic and environmental factors. *SPOCK1* and its gene product tesican-1 showed potential functional relevance to neurodevelopment. Our study has identified a susceptibility gene, *SPOCK1,* which provides novel genetic insights into development of mathematics ability and the basis of human intelligence.

## Methods and Materials

### Participants

We recruited 2,425 grade two to grade six primary students aged 7 to 13 from three counties, Liangshan, Dongming and Cao, in Shandong Province in China. In the first step, Raven’s Progressive Matrices test for nonverbal intelligence was administered to these eligible children individually, whose nonverbal intelligence scores lower than the 25^th^ percentile were excluded from this study. In total, 1622 participants (Liangshan: 501, Dongming: 522, Cao: 599) were eligible for subsequent genotyping and association analysis. Mathematic ability was measured by children’s academic performances of mathematics according to their mid-term and final exam of each semester. The examination papers were designed by education authorities of Shandong Province for each grade respectively according to the curriculum. Therefore, different tests will be applied to children in different grades but children in same grade will take exactly the same test. The tests aimed to evaluate children’s academic performances of mathematics from three perspectives including “understanding numbers”, “computing and knowledge” and “non-numerical processes”. The teacher’s rating process was double-blinded as student’s answer sheet will be randomly and anonymously distributed to different teachers. Answers of all questions are clear and definite. There was no arbitrariness in scoring as teachers from different schools received unified training to ensure that their rating criteria for each student are standardized and objective. The mean score of mid-term and final exam within in same semester that were usually conducted within a 3-month interval was calculated for the analysis. This study was approved by the ethical committee of Tsinghua University School of Medicine. The methods were carried out in accordance with the relevant guidelines. Informed consent was obtained from all subjects.

### Genotyping and quality control in the GWAS

DNA was extracted from blood samples and SNP genotyping was performed with the Affymetrix Axiom Genome-Wide CHB1 and CHB2 arrays (1,284,609 SNPs) by CapitalBio Technology (Beijing, China). Quality control was performed followed by standard quality control metrics^27^. Six samples in Dongming were excluded as they had sex discrepancies between the records and the genetically inferred data, three and four samples in Liangshan and Dongming respectively were excluded as they had overall successful genotyping call rates <95% orhad outlying autosomal heterozygosity rates (out of range of mean ± 3 SD). Next, we removed four and eight individuals in Liangshan and Dongming respectively who had unexpected duplicates or probable relatives (all PI_HAT > 0.20). Finally, we detected population outliers using a method based on the principal component analysis. Common autosomal SNPs in each cohort were employed to identify population outliers in the samples that had passed the quality control, with four original HapMap populations (CEU, CHB, JPT and YRI). In the next step, we performed basic quality control on genotyping data. In total, 40428 and 117542 SNPs in Liangshan and Doming respectively were excluded with call rate of <95%, 57985 and 56307 SNPs in Liangshan and Doming respectively were excluded with minor allele frequency (MAF) of <0.01, 3679 and 2323 SNPs in Liangshan and Doming respectively were excluded with genotype distribution that deviated from the Hardy–Weinberg equilibrium (P < 1.0 × 10^−5^). After quality control procedures had been performed, 494 children with 1182517 SNPs from Liangshan and 504 children with 1108437 SNPSs from Dongming were included in the final analysis.

### Cao sample genotyping

Replication samples were typed at CapitalBio Technology (Beijing, China) with Sequenom MassARRAY platform (San Diego, U.S) according to the manufacturer’s protocol. Briefly, genomic DNA was extracted from saliva of each individual through Oragene^TM^ DNA self-collection kit according to the manufacturer’s instructions (Ottawa, Canada). DNA concentration was determined by Nano Drop 1000 (Waltham, U.S). Specific assays were designed using the MassARRAY Assay Design software package (v3.1). Mass determination was carried out with the MALDI-TOF mass spectrometer and Mass ARRAY Type 4.0 software was used for data acquisition.

### Genome-wide association analysis

After quality control, association analyses and meta analyses were performed using PLINK1.9^28^, fitting an additive model to the data by linear regression model with adjustment for sex, age and principle components in GWAS Liangshan and Dongming samples respectively. SNPs with a P-value < 0.01 were further analyzed by the meta-analysis based method to combine the results from Liangshan and Dongming samples as the discovery phase. SNPs with a P-value < 1.0 × 10^−5^ were selected for replication in Cao population. Finally, a meta-analysis was conducted to combine results from the three populations. A fixed-effect model with inverse variance weighting was used when there was no indication of heterogeneity (P for Cochran’s Q statistic >0.05); otherwise, a random-effect model for the corresponding SNPs was adopted. A Manhattan plot of −log_10_P was generated using the ggplot2 package^29^ in R 2.15.1.

### Power

Power calculations were performed using Quanto version 1.2.4 (http://biostats.usc.edu/Quanto.html). Under the additive model, it had 80% power at the p < 0.05 level to detect association with an allelic variant of 20% frequency accounting for 1.58% and 1.55% of the variance in math score in Liangshan and Dongming populations respectively.

## Additional Information

**How to cite this article**: Chen, H. *et al*. A Genome-Wide Association Study Identifies Genetic Variants Associated with Mathematics Ability. *Sci. Rep.* **7**, 40365; doi: 10.1038/srep40365 (2017).

**Publisher's note:** Springer Nature remains neutral with regard to jurisdictional claims in published maps and institutional affiliations.

## Supplementary Material

Supplement Materials

## Figures and Tables

**Table 1:** Basic characteristics of three populations.

Variables	GWAS		Replication Cao (n = 599)
	Liangshan (n = 494)	Dongming (n = 504)	
Grade			
2	98 (19.84)	98 (19.44)	121 (20.20)
3	103 (20.85)	101 (20.04)	134 (22.37)
4	122 (24.70)	102 (20.24)	136 (22.70)
5	82 (16.60)	99 (19.64)	108 (18.03)
6	89 (18.02)	104 (20.63)	100 (16.69)
Sex			
Male	213 (43.12)	202 (40.08)	283 (47.25)
Female	281 (56.88)	302 (59.92)	316 (52.75)
Age, year	9.79 ± 1.39	9.51 ± 1.44	9.40 ± 1.45
Math score*	97 (93,99)	91 (83, 95)	95 (89,98)

^*^Full score is 100. Data are expressed as number of participants (percentage), mean ± SD or median (Q1, Q3).

**Table 2:** Breakdown of math score in all participants.

	Math score*			*P value*
	<80	80–90	≥90	
**Grade**				
2	12 (6.22)	35 (12.15)	275 (24.64)	<0.001
3	38 (19.69)	91 (31.60)	221 (19.80)	
4	44 (22.80)	47 (16.32)	261 (23.39)	
5	43 (22.28)	70 (24.31)	172 (15.41)	
6	56 (29.02)	45 (15.63)	187 (16.76)	
**Sex**				
Male	77 (39.90)	125 (43.40)	506 (45.34)	0.28
Female	116 (60.10)	163 (56.60)	610 (54.66)	
**Region**				
Liangshan	16 (8.29)	49 (17.01)	429 (38.44)	<0.001
Dongming	92 (47.67)	149 (51.74)	263 (23.57)	
Cao	85 (44.04)	90 (31.25)	424 (37.99)	
Raven score**	45.76 ± 5.63	45.13 ± 5.75	46.69 ± 5.67	<0.001

Data are expressed as number of participants (percentage) or mean ± SD.

^*^Full math score is 100.

^**^Full Raven score is 60.

**Table 3:** SNPs met the criteria in GWAS discovery phase for further replications.

CHR	SNP	MAF	Position	Minor allele	Major allele	Liangshan			Dongming			Meta-analysis*			
						β	SE	P	β	SE	P	β	SE	P**	P_Q_#
1	rs10779824	0.22	230231256	T	C	−1.95	0.53	2.35E-04	−1.91	0.76	1.28E-02	−1.94	0.43	7.79E-06	0.96
1	rs12733302	0.19	230259718	A	G	−2.12	0.55	1.49E-04	−2.19	0.78	5.38E-03	−2.14	0.45	2.18E-06	0.94
3	rs13096852	0.02	99909642	G	A	−5.50	1.58	5.42E-04	−5.52	1.85	2.98E-03	−5.51	1.20	4.50E-06	0.99
4	rs10007531	0.04	31909657	C	T	−4.11	1.07	1.31E-04	−4.67	1.60	3.64E-03	−4.28	0.89	1.37E-06	0.77
4	rs10014657	0.04	31909622	T	G	−4.12	1.07	1.27E-04	−4.96	1.65	2.83E-03	−4.36	0.89	1.09E-06	0.67
5	rs1012694	0.21	136535675	T	C	−2.90	0.56	3.13E-07	−2.36	0.81	3.82E-03	−2.73	0.46	3.17E-09	0.58
5	rs11743006	0.21	136550583	A	C	−2.90	0.56	3.13E-07	−2.27	0.80	4.99E-03	−2.69	0.46	4.33E-09	0.52
5	rs11746206	0.21	136483914	A	G	−2.14	0.56	1.58E-04	−2.47	0.80	2.29E-03	−2.25	0.46	1.08E-06	0.74
5	rs17720840	0.22	136513869	C	T	−2.09	0.57	2.44E-04	−2.27	0.81	5.13E-03	−2.15	0.46	3.46E-06	0.86
5	rs17777541	0.21	136522062	C	G	−2.89	0.56	3.89E-07	−2.28	0.80	4.89E-03	−2.69	0.46	5.23E-09	0.53
5	rs17778739	0.21	136551998	G	C	−2.90	0.56	3.13E-07	−2.28	0.80	4.89E-03	−2.70	0.46	4.22E-09	0.52
5	rs6890599	0.22	136458109	A	G	−1.88	0.55	7.10E-04	−2.29	0.80	4.34E-03	−2.01	0.45	9.31E-06	0.67
6	rs117368522	0.02	109576943	A	G	−6.91	1.80	1.40E-04	−9.86	2.70	2.86E-04	−7.82	1.50	1.77E-07	0.36
6	rs140472982	0.02	65729359	A	G	−5.34	1.63	1.16E-03	−9.76	2.57	1.67E-04	−6.61	1.38	1.63E-06	0.15
6	rs6927133	0.17	49543614	G	A	−1.65	0.59	5.37E-03	−3.56	0.85	3.10E-05	−2.27	0.48	2.61E-06	0.06
7	rs61109185	0.06	149025920	G	T	−4.00	1.05	1.59E-04	−4.37	1.35	1.30E-03	−4.14	0.83	6.04E-07	0.83
9	rs10746628	0.24	82952311	C	T	−1.49	0.53	5.01E-03	−3.26	0.75	1.81E-05	−2.07	0.43	1.60E-06	0.05
11	rs117620298	0.07	17960297	C	T	−3.10	0.90	6.55E-04	−4.17	1.28	1.17E-03	−3.46	0.74	2.77E-06	0.49
13	rs17076102	0.01	52492509	C	T	−6.60	1.81	2.90E-04	−7.27	3.02	1.66E-02	−6.77	1.55	1.26E-05	0.85
15	rs3751566	0.12	22940670	T	C	−2.55	0.68	1.85E-04	−2.52	1.09	2.14E-02	−2.54	0.58	9.94E-06	0.98
15	rs956120	0.13	22937838	G	A	−2.18	0.65	9.26E-04	−3.12	0.95	1.12E-03	−2.48	0.54	4.13E-06	0.42
16	rs1345872	0.18	73500800	G	A	−1.78	0.57	1.95E-03	−2.46	0.85	3.90E-03	−2.00	0.47	2.62E-05	0.51
17	rs11657625	0.16	21381802	C	T	−1.92	0.64	2.92E-03	−3.43	0.83	4.24E-05	−2.48	0.51	9.91E-07	0.15
18	rs12968137	0.08	33796927	T	C	−2.47	0.80	2.31E-03	−4.00	1.17	7.00E-04	−2.96	0.66	8.31E-06	0.28
19	rs74260502	0.04	45101336	T	C	−6.18	1.26	1.23E-06	−4.69	1.54	2.42E-03	−5.58	0.97	9.84E-09	0.45
20	rs11698429	0.30	13009926	T	C	−1.46	0.51	4.32E-03	−2.29	0.68	7.97E-04	−1.76	0.41	1.57E-05	0.32
20	rs3761896	0.32	12989901	C	T	−1.68	0.49	6.63E-04	−2.15	0.67	1.52E-03	−1.84	0.40	3.37E-06	0.57
20	rs3859619	0.32	12993893	C	A	−1.67	0.49	6.76E-04	−2.05	0.67	2.45E-03	−1.80	0.40	5.13E-06	0.65

^*^Meta-analysis of discovery 1 and 2 association results.

^**^P value from fixed model.

^#^P_Q_ is the p value for Cochran’s Q statistic.

**Table 4:** Results of association of 28 SNPs with mathematics ability in replication and combined meta-analysis.

CHR	SNP	Position	Minor allele	Major allele	Cao			Combined Meta*			
					β	SE	*P*	β	SE	P**	*P*_Q_#
1	rs10779824	230231256	T	C	0.64	0.79	4.16E-01	−1.34	0.38	4.26E-04	0.02
1	rs12733302	230259718	A	G	0.21	0.85	8.02E-01	−1.62	0.40	4.99E-05	0.05
3	rs13096852	99909642	G	A	0.73	1.80	6.87E-01	−3.59	1.00	3.27E-04	0.02
4	rs10007531	31909657	C	T	0.31	1.48	8.33E-01	−3.06	0.76	5.59E-05	0.03
4	rs10014657	31909622	T	G	−0.55	1.55	7.22E-01	−3.41	0.78	1.10E-05	0.09
**5**	**rs1012694**	**136535675**	**T**	**C**	**−1.61**	**0.75**	**3.35E-02**	**−2.42**	**0.39**	**6.84E-10**	**0.39**
**5**	**rs11743006**	**136550583**	**A**	**C**	−**1.71**	**0.75**	**2.33E-02**	−**2.43**	**0.39**	**5.67E-10**	**0.44**
5	rs11746206	136483914	A	G	−1.14	0.77	1.40E-01	−1.95	0.39	7.64E-07	0.44
5	rs17720840	136513869	C	T	−1.13	0.76	1.35E-01	−1.87	0.39	2.12E-06	0.51
**5**	**rs17777541**	**136522062**	**C**	**G**	−**1.35**	**0.76**	**7.39E-02**	−**2.33**	**0.39**	**3.13E-09**	**0.27**
**5**	**rs17778739**	**136551998**	**G**	**C**	−**1.63**	**0.76**	**3.24E-02**	−**2.41**	**0.39**	**8.18E-10**	**0.40**
5	rs6890599	136458109	A	G	−0.92	0.75	2.21E-01	−1.72	0.39	9.60E-06	0.42
6	rs117368522	109576943	A	G	−2.58	2.05	2.09E-01	−6.00	1.21	6.97E-07	0.08
6	rs140472982	65729359	A	G	4.12	2.58	1.10E-01	−4.23	1.21	5.07E-04	0.00
6	rs6927133	49543614	G	A	0.93	0.89	2.96E-01	−1.54	0.42	2.85E-04	0.00
7	rs61109185	149025920	G	T	−0.59	1.35	6.63E-01	−3.17	0.71	7.51E-06	0.08
9	rs10746628	82952311	C	T	−0.23	0.75	7.61E-01	−1.61	0.37	1.63E-05	0.02
11	rs117620298	17960297	C	T	0.48	1.53	7.53E-01	−2.72	0.66	4.39E-05	0.05
13	rs17076102	52492509	C	T	2.32	2.09	2.69E-01	−3.57	1.25	4.17E-03	0.00
15	rs3751566	22940670	T	C	−0.22	0.89	8.05E-01	−1.85	0.48	1.26E-04	0.09
15	rs956120	22937838	G	A	−0.17	0.88	8.42E-01	−1.85	0.46	5.84E-05	0.06
16	rs1345872	73500800	G	A	−0.31	0.79	6.94E-01	−1.55	0.41	1.40E-04	0.15
17	rs11657625	21381802	C	T	1.05	0.91	2.53E-01	−1.65	0.44	2.01E-04	0.00
18	rs12968137	33796927	T	C	2.85	1.09	9.14E-03	−1.39	0.57	1.45E-02	0.00
19	rs74260502	45101336	T	C	0.93	2.03	6.49E-01	−4.37	0.88	6.46E-07	0.01
20	rs11698429	13009926	T	C	0.87	0.68	1.99E-01	−1.06	0.35	2.30E-03	0.00
20	rs3761896	12989901	C	T	0.75	0.67	2.60E-01	−1.17	0.34	6.12E-04	0.00
20	rs3859619	12993893	C	A	0.79	0.67	2.40E-01	−1.14	0.34	8.23E-04	0.00

^*^Meta-analysis of all three populations.

^**^P value from fixed model.

^#^P_Q_ is the p value for Cochran’s Q statistic.

**Table 5:** Summary of GWA scan and replication studies for the significant SNPs.

SNP	Stage	β	SE	*P*	*P*_Q_#
rs1012694 T/C* chr5:136535675**	Discovery 1	−2.90	0.56	3.13E-07	
	Discovery 2	−2.36	0.81	3.82E-03	
	Discovery combine	−2.66	0.49	8.41E-08	
	Discovery meta	−2.73	0.46	3.17E-09	0.58
	Replication	−1.61	0.75	3.35E-02	
	**Combined meta**	−**2.42**	**0.39**	**6.84E-10**	**0.39**
rs11743006 A/C* chr5:136550583**	Discovery 1	−2.90	0.56	3.13E-07	
	Discovery 2	−2.27	0.80	4.99E-03	
	Discovery combine	−2.61	0.49	1.28E-07	
	Discovery meta	−2.69	0.46	4.33E-09	0.52
	Replication	−1.71	0.75	2.33E-02	
	**Combined meta**	−**2.43**	**0.39**	**5.67E-10**	**0.44**
rs17778739 G/C* chr5:136551998**	Discovery 1	−2.90	0.56	3.13E-07	
	Discovery 2	−2.28	0.80	4.89E-03	
	Discovery combine	−2.62	0.49	1.18E-07	
	Discovery meta	−2.70	0.46	4.22E-09	0.52
	Replication	−1.63	0.76	3.24E-02	
	**Combined meta**	−**2.41**	**0.39**	**8.18E-10**	**0.40**
rs17777541 C/G* chr5:136522062**	Discovery 1	−2.89	0.56	3.89E-07	
	Discovery 2	−2.28	0.80	4.89E-03	
	Discovery combine	−2.61	0.49	1.25E-07	
	Discovery meta	−2.69	0.46	5.23E-09	0.53
	Replication	−1.35	0.76	7.39E-02	
	**Combined meta**	−**2.33**	**0.39**	**3.13E-09**	**0.27**

Discovery 1: Liangshan population.

Discovery 2: Dongming population.

Discovery combine: Liangshan and Dongming populations combined directly.

Discovery meta: meta-analysis of discovery 1 and 2 association results.

Replication: Cao population.

Combined meta: meta-analysis of association results in three populations.

^*^Minor allele/Major allele.

^**^Chromosomalposition (Build 37).

^#^P value for Cochran’s Qstatistic.

## References

[b1] Kucian K. & von Aster M. Developmental dyscalculia. Eur. J. Pediatr. 174, 1–13 (2015). 25529864 10.1007/s00431-014-2455-7

[b2] Ritchie S. J. & Bates T. C. Enduring links from childhood mathematics and reading achievement to adult socioeconomic status. Psychol. Sci. 24, 1301–8 (2013). 23640065 10.1177/0956797612466268

[b3] McCarthy M. I. . Genome-wide association studies for complex traits: consensus, uncertainty and challenges. Nat. Rev. Genet. 9, 356–69 (2008). 18398418 10.1038/nrg2344

[b4] Docherty S. J. . A genome-wide association study identifies multiple loci associated with mathematics ability and disability. Genes. Brain. Behav. 9, 234–47 (2010). 20039944 10.1111/j.1601-183X.2009.00553.x PMC2855870

[b5] Baron-Cohen S. . A genome wide association study of mathematical ability reveals an association at chromosome 3q29, a locus associated with autism and learning difficulties: a preliminary study. PLoS One 9, e96374 (2014). 24801482 10.1371/journal.pone.0096374 PMC4011843

[b6] Davis O. S. P. . The correlation between reading and mathematics ability at age twelve has a substantial genetic component. Nat. Commun. 5, 4204 (2014). 25003214 10.1038/ncomms5204 PMC4102107

[b7] Hart S. A. . Exploring how symptoms of attention-deficit/hyperactivity disorder are related to reading and mathematics performance: general genes, general environments. Psychol. Sci. 21, 1708–15 (2010). 20966487 10.1177/0956797610386617 PMC3708699

[b8] Baron-Cohen S., Wheelwright S., Burtenshaw A. & Hobson E. Mathematical Talent is Linked to Autism. Hum. Nat. 18, 125–31 (2007). 26181845 10.1007/s12110-007-9014-0

[b9] Ludwig K. U. . A common variant in Myosin-18B contributes to mathematical abilities in children with dyslexia and intraparietal sulcus variability in adults. Transl. Psychiatry 3, e229 (2013). 23423138 10.1038/tp.2012.148 PMC3591001

[b10] Pettigrew K. A. . Lack of replication for the myosin-18B association with mathematical ability in independent cohorts. Genes. Brain. Behav. 14, 369–376 (2015). 25778778 10.1111/gbb.12213 PMC4672701

[b11] Semenza C. . Genetics and mathematics: FMR1 premutation female carriers. Neuropsychologia 50, 3757–63 (2012). 23123760 10.1016/j.neuropsychologia.2012.10.021

[b12] Carvalho M. R. S. . Are 22q11.2 distal deletions associated with math difficulties? Am. J. Med. Genet. A 164A, 2256–62 (2014). 24989330 10.1002/ajmg.a.36649

[b13] Davis J. M. . DUF1220 copy number is linearly associated with increased cognitive function as measured by total IQ and mathematical aptitude scores. Hum. Genet. 134, 67–75 (2015). 25287832 10.1007/s00439-014-1489-2 PMC5898241

[b14] Edgell C.-J. S., BaSalamah M. A. & Marr H. S. Testican-1: a differentially expressed proteoglycan with protease inhibiting activities. Int. Rev. Cytol. 236, 101–22 (2004). 15261737 10.1016/S0074-7696(04)36003-1

[b15] Cheng W., Su Y. & Xu F. CHD1L: a novel oncogene. Mol. Cancer 12, 170 (2013). 24359616 10.1186/1476-4598-12-170 PMC3931672

[b16] Li Y. . SPOCK1 is regulated by CHD1L and blocks apoptosis and promotes HCC cell invasiveness and metastasis in mice. Gastroenterology 144, 179–191 e4 (2013). 23022495 10.1053/j.gastro.2012.09.042

[b17] Shu Y.-J. . SPOCK1 as a potential cancer prognostic marker promotes the proliferation and metastasis of gallbladder cancer cells by activating the PI3K/AKT pathway. Mol. Cancer 14, 12 (2015). 25623055 10.1186/s12943-014-0276-y PMC4320842

[b18] Miao L. . SPOCK1 is a novel transforming growth factor-β target gene that regulates lung cancer cell epithelial-mesenchymal transition. Biochem. Biophys. Res. Commun. 440, 792–7 (2013). 24134845 10.1016/j.bbrc.2013.10.024

[b19] Song X. . Up-regulation of SPOCK1 induces epithelial-mesenchymal transition and promotes migration and invasion in esophageal squamous cell carcinoma. J. Mol. Histol. 46, 347–56 (2015). 26077618 10.1007/s10735-015-9627-2

[b20] Kim H.-P. . Testican-1-mediated epithelial-mesenchymal transition signaling confers acquired resistance to lapatinib in HER2-positive gastric cancer. Oncogene 33, 3334–41 (2014). 23873022 10.1038/onc.2013.285

[b21] Dhamija R., Graham J. M., Smaoui N., Thorland E. & Kirmani S. Novel de novo SPOCK1 mutation in a proband with developmental delay, microcephaly and agenesis of corpus callosum. Eur. J. Med. Genet. 57, 181–4 (2014). 24583203 10.1016/j.ejmg.2014.02.009

[b22] Marr H. S. & Edgell C. J. S. Testican-1 inhibits attachment of Neuro-2a cells. Matrix Biol. 22, 259–66 (2003). 12853036 10.1016/s0945-053x(03)00036-2

[b23] Bocock J. P., Edgell C.-J. S., Marr H. S. & Erickson A. H. Human proteoglycan testican-1 inhibits the lysosomal cysteine protease cathepsin L. Eur. J. Biochem. 270, 4008–15 (2003). 14511383 10.1046/j.1432-1033.2003.03789.x

[b24] Röll S., Seul J., Paulsson M. & Hartmann U. Testican-1 is dispensable for mouse development. Matrix Biol. 25, 373–81 (2006). 16806869 10.1016/j.matbio.2006.05.004

[b25] Charbonnier F. . Expression of the proteoglycan SPOCK during mouse embryo development. Mech. Dev. 90, 317–321 (2000). 10640720 10.1016/s0925-4773(99)00255-5

[b26] Iseki K. . Altered expression pattern of testican-1 mRNA after brain injury. Biomed. Res. 32, 373–8 (2011). 22199127 10.2220/biomedres.32.373

[b27] Anderson C. A. . Data quality control in genetic case-control association studies. Nat. Protoc. 5, 1564–73 (2010). 21085122 10.1038/nprot.2010.116 PMC3025522

[b28] Purcell S. . PLINK: a tool set for whole-genome association and population-based linkage analyses. Am. J. Hum. Genet. 81, 559–75 (2007). 17701901 10.1086/519795 PMC1950838

[b29] Wickham H. Ggplot2: Elegant Graphics for Data Analysis (eds Gentleman R. .) 180–185 (Springer, 2009).

