# Erratum: A Genome-Wide Association Study Identifies Genetic Variants Associated with Mathematics Ability

**DOI:** 10.1038/srep44512

**Published:** 2017-04-11

**Authors:** Huan Chen, Xiao-hong Gu, Yuxi Zhou, Zeng Ge, Bin Wang, Wai Ting Siok, Guoqing Wang, Michael Huen, Yuyang Jiang, Li-Hai Tan, Yimin Sun

Scientific Reports 7: Article number: 4036510.1038/srep40365; published online: 02 03 2017; updated: 04 11 2017

This Article contains errors in Table 2. The correct Table 2 appears below:

## Figures and Tables

**Table 2:** 

	Math score*			P value
	<80	80–90	≥90	
Grade				
2	10 (5.41)	36 (12.54)	271 (24.09)	<0.001
3	37 (20)	89 (31.01)	212 (18.84)	
4	44 (23.78)	43 (14.98)	273 (24.27)	
5	42 (22.7)	73 (25.44)	174 (15.47)	
6	52 (28.11)	46 (16.03)	195 (17.33)	
Sex				
Male	72 (38.92)	121 (42.16)	505 (44.89)	0.27
Female	113 (61.08)	166 (57.84)	620 (55.11)	
Region				
Liangshan	18 (9.73)	49 (17.07)	427 (37.96)	<0.001
Dongming	92 (49.73)	149 (51.92)	263 (23.38)	
Cao	75 (40.54)	89 (31.01)	435 (38.67)	
Raven score**	45.76 ± 5.63	45.13 ± 5.75	46.69 ± 5.67	<0.001

