# Supplementary material for: A Genome-Wide Association Study Identifies Genetic Variants Associated with Mathematics Ability
Source: Sci Rep. 2017 Feb 3;7:40365. doi: 10.1038/srep40365 (PMC5290743; doi:10.1038/srep40365)
Supplement: Supplement Materials [file srep40365-s1.doc]

**A** **Genome-Wide Association Study Identifies Genetic Variants Associated with Mathematics Ability**

Huan Chen^1,2*^, Xiao-hong Gu^3*^, Yuxi Zhou^4,5*^, Zeng Ge^4,5*^, Bin Wang^4,5^, Wai Ting Siok^6^, Guoqing Wang^4,5^, Michael Huen^7^, Yuyang Jiang^8^, Li-Hai Tan^1,9**^, Yimin Sun^4,5,8,10**^

^1^Center for Neurogenetics, Shenzhen Institute of Neuroscience, Shenzhen, 518057, China

^2^State Key Laboratory of Proteomics, Beijing Proteome Research Center, Beijing Institute of Radiation Medicine, Beijing, 102206, China

^3^Department of Healthy Management, Research Institute of Surgery, DaPing Hospital, Third Military Medical University, Chongqing, 400042, China

^4^CapitalBio eHealth Science & Technology (Beijing) Co., Ltd., Beijing, 102206, China

^5^National Engineering Research Center for Beijing Biochip Technology, Beijing, 102206, China

^6^Department of Linguistics, The University of Hong Kong, Hong Kong, China

^7^Department of Anatomy, The University of Hong Kong, Hong Kong, China

^8^The State Key Laboratory Breeding Base-Shenzhen Key Laboratory of Chemical Biology, The Graduate School at Shenzhen, Tsinghua University, Shenzhen, 518055, China

^9^School of Biomedical Engineering, Shenzhen University Health Science Center, Shenzhen, 518060, China

^10^Department of Biomedical Engineering, Medical Systems Biology Research Center, Tsinghua University School of Medicine, Beijing, 100084, China

*These authors contributed equally to this work.

**Correspondence should be addressed to Yimin Sun ([ymsun@capitabio.com](mailto:ymsun@capitabio.com)) or to Li-Hai Tan ([tanlh@szu.edu.cn](mailto:tanlh@szu.edu.cn)).

Supplementary Information


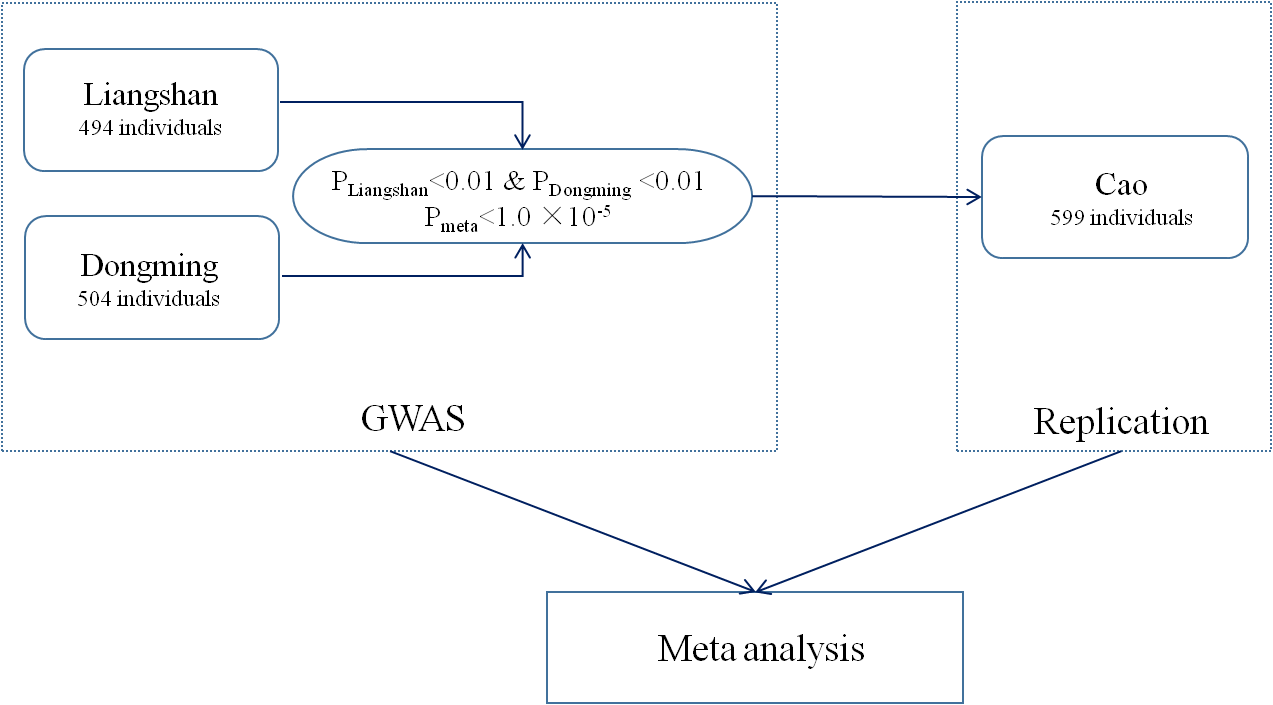


Supplementary Figure S1 Summary of study design


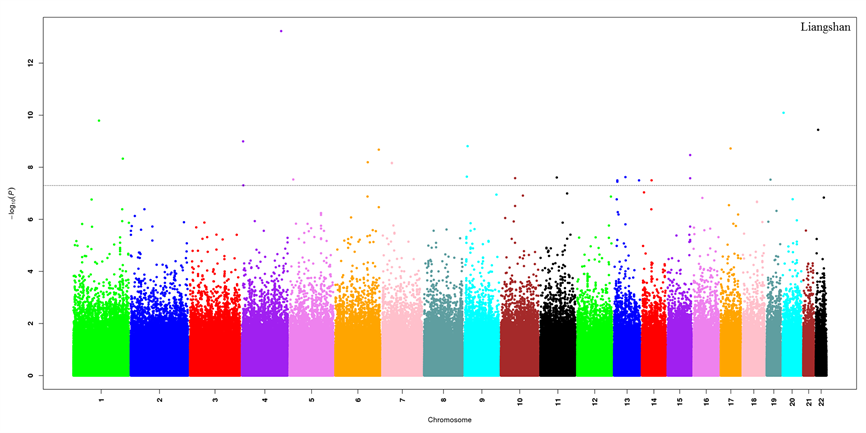


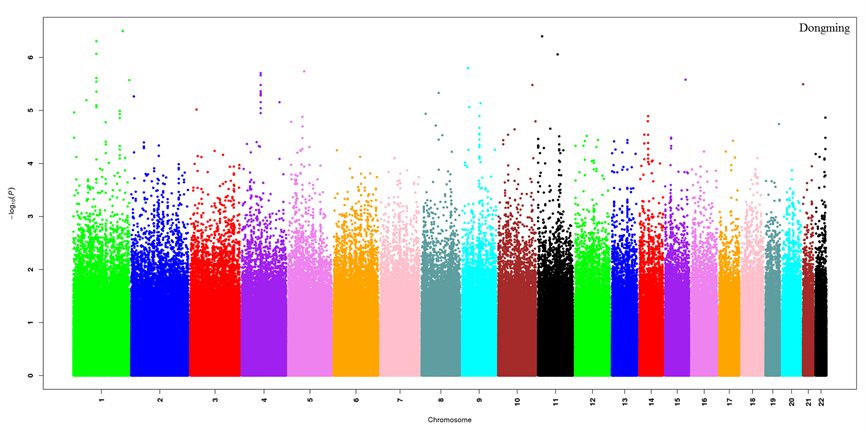


Supplementary Figure S2. Manhattan plot of –log_10_ (P values) of Liangshan population and Dongming population from the additive model after adjustment for sex, age and nominal significant principal components in GWAS. 13082 and 11170 SNPs had P value <0.01 in Liangshan and Dongming population respectively.


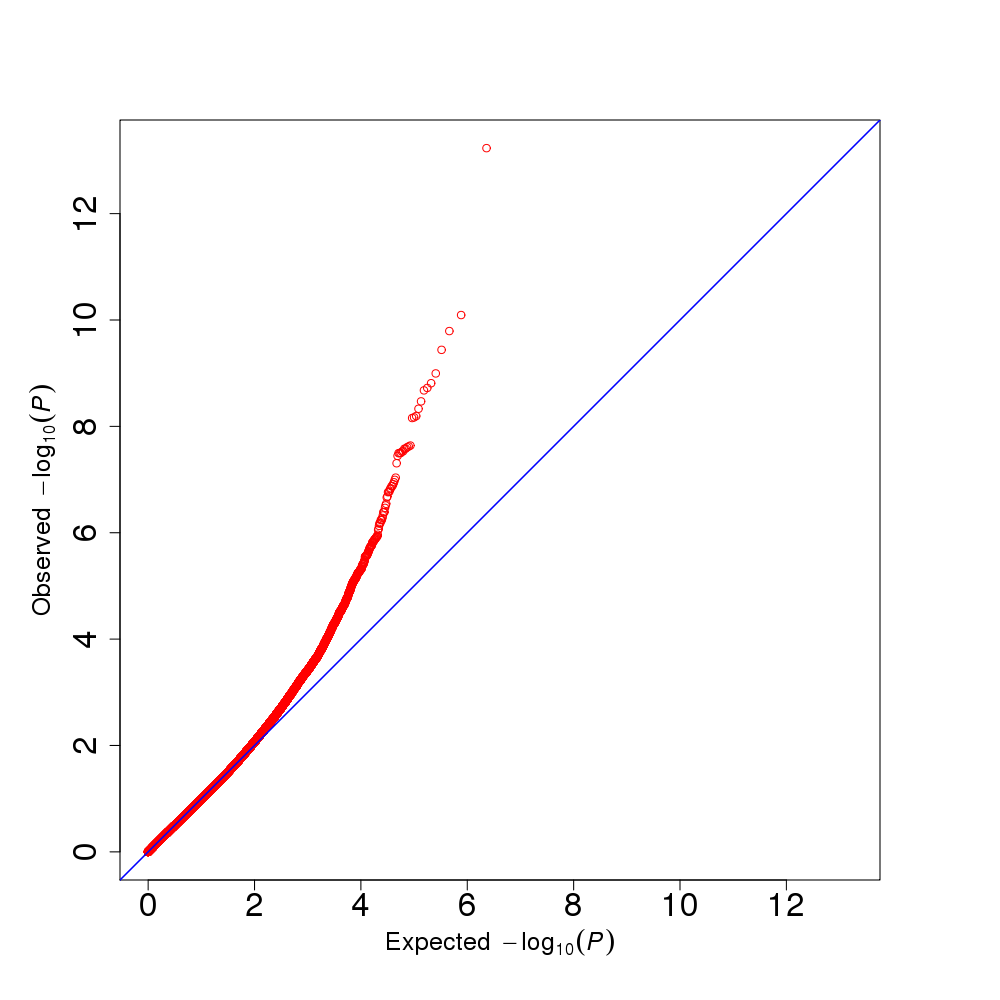

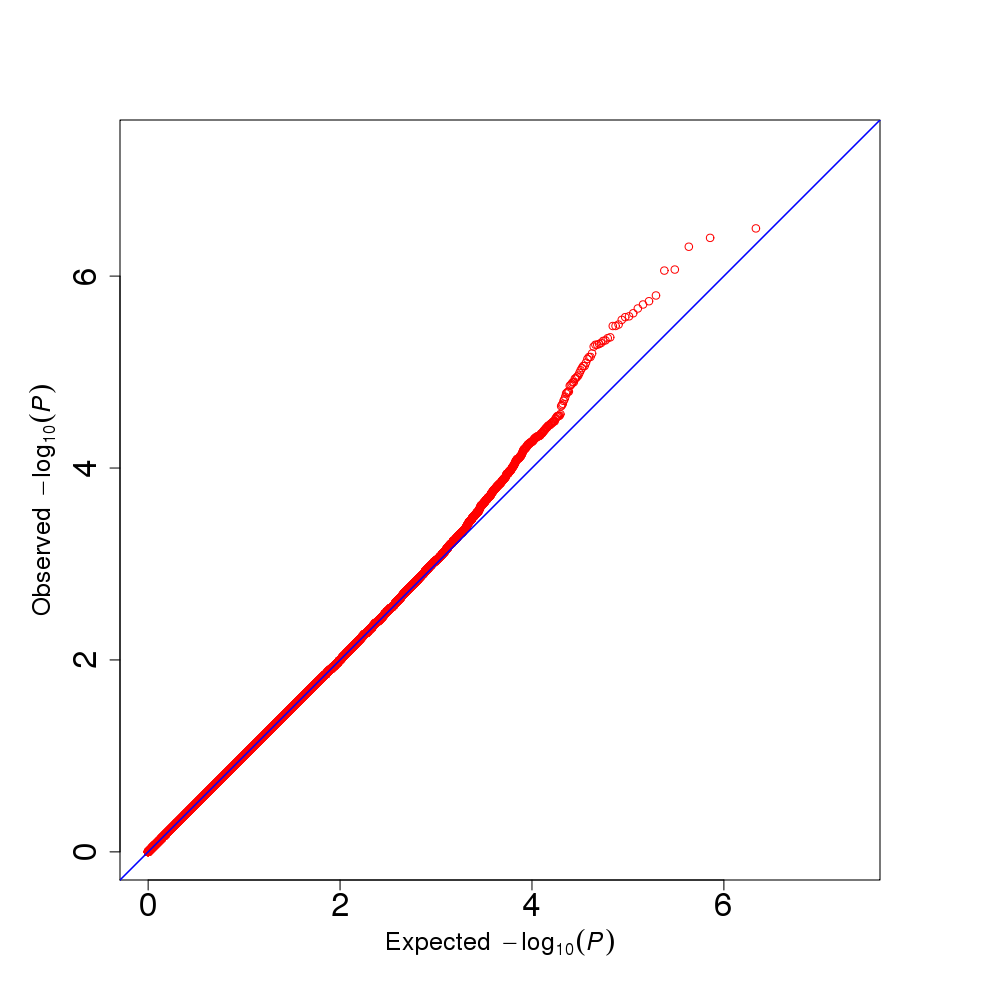


Supplementary Figure S3. Quantile-Quantile plots for the two discovery populations, Liangshan (the left panel) and Dongming (the right panel).
